# Personal

**Published:** 1891-03

**Authors:** 


### Personal.

Dr. L. S. McMurtry, of Louisville, Ky., who was elected President of the Southern Surgical and Gynecological Association, meeting in Atlanta, November, 1890, is a native of Kentucky and a graduate of Centre College in that State. He graduated in medicine at the University of Louisiana, and afterwards studied at the University of New York. He was Professor of Anatomy in the Kentucky School of Medicine several years, and is now surgeon of the Sts. Mary and Elizabeth Hospital in that city. Two years since, he was President of the Kentucky State Medical Society. Dr. McMurtry has made numerous contributions to medical literature, relating mostly to abdominal surgery and the diseases of women. He has been elected a Fellow of the Obstetrical Society of Edinburgh. He is about thirty-eight years of age.—*The Dixie Doctor.*

Dr. Charles A. L. Reed, of Cincinnati, who was elected Chairman of the Section of Obstetrics and Diseases of Women, at the Nashville meeting of the American Medical Association, will preside over his Section at the meeting to be held in Washington, D. C., May 5, 6, 7, and 8, 1891. Dr. Reed is one of the foremost men in his department of the science and art of medicine, and will undoubtedly make his talents as an executive officer felt in the work done by his Section this year. He is desirous that all who wish to read papers relating to Obstetrics, Gynecology, or Abdominal Surgery, may furnish their titles at an early day. His address is 311 Elm street, Cincinnati, O.

